# MyBASE: a database for genome polymorphism and gene function studies of *Mycobacterium*

**DOI:** 10.1186/1471-2180-9-40

**Published:** 2009-02-20

**Authors:** Xinxing Zhu, Suhua Chang, Kechi Fang, Sijia Cui, Jun Liu, Zuowei Wu, Xuping Yu, George F Gao, Huanming Yang, Baoli Zhu, Jing Wang

**Affiliations:** 1 James D Watson Institute of Genome Sciences of Zhejiang University, Hangzhou 310007, PR China; 2 Behavioral Genetics Center, Institute of Psychology, Chinese Academy of Sciences, Beijing 100101, PR China; 3 Department of Molecular Genetics, University of Toronto, 1 King's College Circle, Toronto, Ontario, Canada; 4 Institute of Microbiology, Chinese Academy of Sciences, Beijing 100101, PR China; 5 Institute of Preventive Veterinary Medicine, College of Animal Sciences, Zhejiang University, Hangzhou 310029, PR China; 6 Beijing Institute of Genomics, Chinese Academy of Sciences, Beijing 100029, PR China

## Abstract

**Background:**

Mycobacterial pathogens are a major threat to humans. With the increasing availability of functional genomic data, research on mycobacterial pathogenesis and subsequent control strategies will be greatly accelerated. It has been suggested that genome polymorphisms, namely large sequence polymorphisms, can influence the pathogenicity of different mycobacterial strains. However, there is currently no database dedicated to mycobacterial genome polymorphisms with functional interpretations.

**Description:**

We have developed a **my**cobacterial data**base **(MyBASE) housing genome polymorphism data and gene functions to provide the mycobacterial research community with a useful information resource and analysis platform. Whole genome comparison data produced by our lab and the novel genome polymorphisms identified were deposited into MyBASE. Extensive literature review of genome polymorphism data, mainly large sequence polymorphisms (LSPs), operon predictions and curated annotations of virulence and essentiality of mycobacterial genes are unique features of MyBASE. Large-scale genomic data integration from public resources makes MyBASE a comprehensive data warehouse useful for current research. All data is cross-linked and can be graphically viewed via a toolbox in MyBASE.

**Conclusion:**

As an integrated platform focused on the collection of experimental data from our own lab and published literature, MyBASE will facilitate analysis of genome structure and polymorphisms, which will provide insight into genome evolution. Importantly, the database will also facilitate the comparison of virulence factors among various mycobacterial strains. MyBASE is freely accessible via http://mybase.psych.ac.cn.

## Background

Mycobacteria are notorious for its two species, *Mycobacterium tuberculosis *(*M. tb*) and *Mycobacterium leprae *(*M. leprae*), the causative agent of tuberculosis (TB) and leprosy, respectively. In addition to *M. tb *and *M. leprae*, a number of mycobacterial pathogens also cause human and animal diseases, including *Mycobacterium bovis *(*M. bovis*), the causative agent of classical bovine tuberculosis, and *Mycobacterium ulcerans *(*M. ulcerans*), which causes Buruli ulcers. The emergence of multi-drug resistant strains of pathogenic mycobacteria has made the development of better vaccines and new drugs and novel control strategies a top priority.

The genome of *M. tb *H37Rv was the first mycobacterial genome to be sequenced and was published in 1998 [1], which was followed by the genome of *M. leprae *in 2001 [2]. The complete sequencing of these genomes greatly contributed to the understanding of the unique physiology and pathogenesis of mycobacteria. With the development of DNA sequencing technologies in recent years, a total of 18 complete mycobacterial genomes have been available and deposited in public domains thus far. This progress offers an unprecedented opportunity to understand the virulence mechanisms of mycobacteria at the molecular level, which offers insight into the development of potential control strategies.

One of the most significant findings in mycobacterial research was from the genome-wide comparison between virulent (e.g. *M. tb *H37Rv or *M. bovis*) and avirulent strains (e.g. *M. bovis *BCG) [3]. This genomic comparison unveiled large sequence polymorphisms (LSPs), usually called regions of difference (RDs), which are believed to be the major source of genomic diversity [4,5] and probably contribute to the phenotypic differences [6]. Some of the LSPs/RDs have been shown be important for virulence and pathogenicity. For example, RD1, which is deleted in all BCG strains but is present in virulent strains of *M. tb *or *M. bovis*, has been shown to be essential for *M. tb *virulence [7-9]. The success of systematic genetic screening of mycobacterial mutants from different environments [10-13], coupled with focused investigation into individual virulence genes, has contributed to the functional genomic data of mycobacteria [14], which has provided useful information in understanding the physiology and pathogenesis of this unique bacterial genus.

Currently, several public resources for mycobacterial research are available, including the TB database [15], which is an integrated platform of genomic data with special interest in microarray analysis; GenoList http://genolist.pasteur.fr/, which focuses on the gene annotation of six mycobacterial strains [16]; MycoDB from xBASE [17,18], which provides search and visualization tools for genome comparison of mycobacteria; MycoperonDB [19], which is a database of predicted operons in 5 mycobacterial species; MGDD [20], a mycobacterial genome divergence database derived from an anchor-based comparison approach [21]; GenoMycDB [22], a database for pair-wise comparison of six mycobacterial genomes; and MtbRegList [23], which is dedicated to the analysis of transcriptional regulation of *M. tb*. Although each of these databases provides unique and useful information, none are focused on LSPs, essential genes, and the relationship between these and virulence. MyBASE was therefore developed to meet these needs. In addition to providing a platform for analyzing all published mycobacterial genomes, MyBASE features important information on genomic polymorphisms, virulence genes, and essential genes. As such, MyBASE will help researchers to easily explore and analyze data in a user-friendly and cross-referenced fashion, thereby facilitating functional genomic studies. This will inevitably enhance our understanding on the virulence mechanisms, genome structure, and molecular evolution of mycobacteria.

## Construction and content

### Data sources and curation

MyBASE contains data from both our own experiments and public resources. There are four main types of data: 1) genome sequences with curated annotations, 2) genome polymorphism data, particularly LSPs identified among different mycobacterial genomes, 3) functional gene annotations with a specific focus on virulence genes and essential genes, and 4) predicted operons.

All complete genome sequences and original annotation files were downloaded from NCBI ftp://ftp.ncbi.nih.gov/genomes/Bacteria. Curations were made to clarify inconsistencies resulting from different annotations provided by the original sequence providers. For Clusters of Orthologous Groups (COGs) that were inconsistently designated [24], we refined the COGs using the algorithm previously described [25].

We have recently used the NimbleGen tiling microarray method for whole-genome comparison of 13 BCG strains with subsequent confirmation by DNA re-sequencing [26]. A total of 42 deletions were identified, four of which are novel [26]. These novel deletions are believed to have an impact on virulence or immunogenicity of the corresponding BCG strains [26]. All data and analytical results were incorporated into MyBASE. In addition to our self-generated data, other polymorphism datasets, particularly LSPs/RDs that were included in MyBASE were extracted from public literatures. After the first usage of microarray to study genome polymorphism in 1999 [3], a growing trend emerged to generate systematic genome polymorphism data [27-29]. We performed an extensive literature review to extract information about each LSP/RD from original experiments. We found inconsistencies between the nomenclature of LSPs (RDs) used by different groups and so to avoid further confusion, we have kept the original nomenclature from each group. However, we have provided the reference information and a hyperlink to the PubMed entry for each LSP/RD dataset.

Virulence, essentiality and other functional annotations of mycobacterial genes were extracted and corrected through data mining of public resources [10-14,30]. Virulence of mycobacterial genes was evaluated by phenotypic outcomes observed from animal and cellular models of *M. tb *infections (e.g., mouse, guinea pig, macrophages, etc.) for the corresponding mutants [14]. Recently, with the success of genetic manipulation of mycobacterial genes, a number of new virulence factors have been uncovered [31-35]. Since the role of some of these genes in pathogenesis are still in dispute [36,37], the annotations of experimental evidence for virulence have been provided to facilitate further investigation.

Operons of mycobacterial genomes were predicted using methods described by Alm *et al*. [38]. This method combines a comparative genomic approach with genome-specific distance models, and has shown some improvements in operon prediction [39].

### System design and implementation

MyBASE was developed using our established pipeline for biological databases [40-44]. It consists of three hardware components: a World Wide Web server, a database server, and a server for sequence analysis. The system is based on a MySQL relational database and the front end consists of a set of JSP scripts running on a Tomcat web server. Hibernate, a high-performance object/relational persistence and query service for Java, was used for system development. The search engine, Multi-genome Comparison Viewer, was developed using Java. Genome Viewer was implemented using CGView [45].

## Utility and discussion

### Database usage and the toolbox

All the data in MyBASE can be easily explored using the toolbox. The keyword-based search engine enables a multiple keyword (e.g. gene name, COG number, etc.) search across MyBASE, while the BLAST-based sequence search engine allows user to quickly find similar genes to the query.

**Figure 1:**
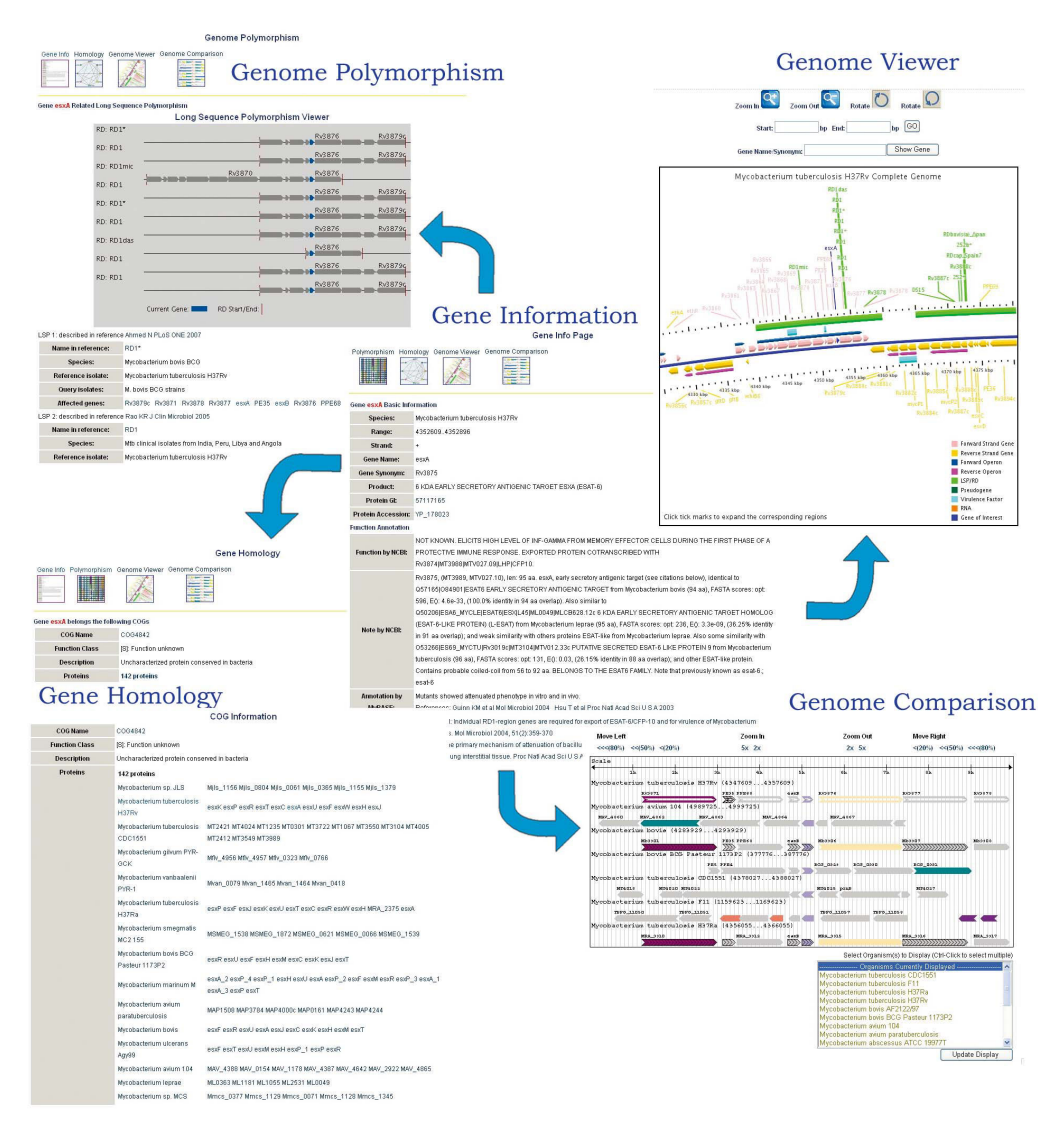
**Schematic representation of the data repository and the interrelation of functional modules in MyBASE**. After the gene of interest is found, users can check whether it is in a genomic polymorphic region, compare the selected genome with MCV, explore the details of its genome segment with Genome Viewer or view its homolog distributions.

LSP/RD data is a distinct feature of MyBASE. The Polymorphism-LSP/RD module was developed to explore and mine the LSP/RD datasets. Users can search for a genomic polymorphism region by its name (e.g. RD1), the name of reference strain and query strain in the experiment, start/end positions within its genome, or by literature information. Users can also visualize the distributions of selected RDs in the genome by using LSP/RD Viewer. RDs in the same dataset are present in one solid line according to its position along the genome (upper-left in Figure 1). Experimental information can be seen when users mouse over the LSP/RD region. To keep the data content in MyBASE most up-to-date, the "LSP/RD upload" module was designed for the user to upload their own LSP/RD data to MyBASE.

The Multi-genome Comparison Viewer (MCV) allows users to rapidly align and compare mycobacterial genome synteny by selecting an anchor gene of interest. This module is helpful for genome structure and evolutionary analysis of mycobacteria. Users can select any number of genomes, zoom in or out and move upstream or downstream along the genome in the viewer. Genes in MCV with the same color-coding are predicted homologs via COG designation, while grey indicates that no homolog was detected. More importantly, MCV also displays various featured annotations in MyBASE with different legends. Virulence factors, pseudogenes, and genes in an operon or polymorphic region are all presented in this graphic way. By clicking the gene, users can either re-anchor the viewer with this gene or navigate to the detailed gene information page.

Genome Viewer allows users to explore individual genomes with customized featured annotations, which include operons, LSPs/RDs, pseudogenes, and virulence factors. In addition, users can visualize a particular segment of a genome by zooming in/out, rotating or defining the start and end positions.

All data and tools in MyBASE are cross-linked. Users can start from searching a particular gene, for example, *esxA*, which is a virulence determinant that encodes a secretory protein [6,46,47], and then search each functional module, including polymorphisms (LSPs/RDs) for related LSP information. Furthermore, MCV and Genome Viewer can be used to compare the genome structure among selected genomes and to check other genomic features within the corresponding segment, respectively. Using these tools, we can see that *esxA *is located in RD1 and that its functional properties are represented by different legends. Users may also begin from a polymorphism search and then navigate to a gene page, MCV or Genome Viewer. Overall, MyBASE forms a highly-integrated and inter-correlated platform for efficient utilization and exploration of functional and comparative genomic data (Figure 1).

### Future developments

The goal of MyBASE is to provide the mycobacterial research community with a useful resource and analysis platform for the functional and evolutionary investigation of mycobacteria. Newly generated genomic data and functional annotations by the research community will be added to MyBASE periodically to keep the database up-to-date. The functionality of the LSP search and viewer will be enriched and enhanced. In addition, new tools, such as software packages for phylogenomic study will be integrated. Finally, MyBASE also provides an opportunity for the mycobacterial research community to standardize nomenclature, data formats of gene, and polymorphism annotations.

## Conclusion

MyBASE is a unique data warehouse and analysis platform for the mycobacterial research community, which features a collection and curation of a large amount of LSP and functional genomic data. By developing various tools, MyBASE can help researchers to easily explore and investigate genome deletions, virulence factors, essential genes, and operon structure of mycobacteria.

## Availability and requirements

The database is freely available on http://mybase.psych.ac.cn.

## Authors' contributions

XZ designed the database, collected, curated the data and wrote the manuscript. SC analyzed the data and developed the database. KF and SC developed the database and did the programming work. JL, ZW, and XY performed the microarray experiments and analyzed the data. GFG, and HY revised the manuscript. BZ and JW supervised the work, manage the team and wrote the manuscript. All authors read and approved the final manuscript.
